# Recent advances in the diagnosis and management of gestational diabetes

**DOI:** 10.4274/tjod.71677

**Published:** 2014-09-15

**Authors:** Pınar Solmaz Hasdemir, Hasan Terzi, Faik Mümtaz Koyuncu

**Affiliations:** 1 Celal Bayar University Faculty of Medicine, Department of Obstetrics and Gynecology, Manisa, Turkey; 2 Kocaeli Derince Education and Research Hospital, Clinic of Obstetrics and Gynecology, Kocaeli, Turkey

**Keywords:** gestational diabetes, glucose tolerance test, oral antidiabetic agents

## Abstract

Gestational diabetes is a condition which is seen in 7% of pregnancies and have potential risks for both mother and fetus. Despite its importance, there is not any golden standard approaches to the diagnosis and management of the disease. The aim of this review was to investigate the advances in the diagnosis and management of gestational diabetes in recent years.

## INTRODUCTION

The glucose intolerance occurring for the first time or diagnosed during pregnancy is referred to as gestational diabetes mellitus (GDM). Of all pregnancies, 7% are complicated with GDM (ranging from 1% to 14% depending on the population studies and diagnostic tests employed) the rate of which differs from one country to another, and this rate corresponds to 200.000 cases per year^(1)^. In addition to increased perinatal mortality and morbidity, intrauterine hyperglycemia results in an increased risk of obesity, metabolic and cardiovascular disorders, and malignancy in future life of fetus after delivery^(2)^. Most of the above-mentioned changes recover after delivery; however, it is also likely that these changes may persist after delivery. With regards to general distribution of obesity and diabetes in the population, it is more likely for women with childbearing potential to have type 2 diabetes mellitus, and the rate of women with unrecognized type 2 diabetes is increasing among pregnancy women.

The diagnostic criteria for GDM were first described 40 years age; however, there is no single screening method complying with the international standards. It is obvious that there is a great demand for a uniform strategy in the diagnosis and classification of GDM, the challenges to adopt such strategy is well-known and discussed for years. The tests that have been used until recently have mostly focused on the risk of developing diabetes in future life after pregnancy and not on perinatal outcomes of GDM^(3)^.

## DIAGNOSTIC CRITERIA FOR GESTATIONAL DIABETES

GDM brings some risks to the mother and newborn baby. The Hyperglycemia and Adverse Pregnancy Outcomes (HAPO) study published in 2008 has caused questioning of conventional method used in the diagnosis of GDM. In this epidemiological study, a total of 25.505 pregnant women from 15 centers in 9 countries and between 24 and 36 weeks of gestation were examined with 75 gr OGTT, and the probability of maternal, fetal, and neonatal side effects was shown to have increased in relation to glycemic status between at 24-28 weeks of gestation (even with blood glucose levels previously regarded as normal)^(4)^. The study concluded that maternal blood glucose level, even if it was lower than the threshold required for the diagnosis of GDM, was found to be associated with increased birth weight and cord blood C-peptide levels, and these results prompted the researchers to revise diagnostic criteria for gestational diabetes. The study group recommended 75 grams OGTT to be performed in all pregnant women between 24-28 weeks of gestation. This also pointed to the need to change the threshold for plasma glucose level while fasting and at 1 hour and 2 hours to diagnose GDM.

After having extensive discussions between years 2008 and 2009, the International Association of the Diabetes and Pregnancy Study Groups (IADPSG) declared a consensus report with the participation of 220 delegates from 40 countries. Accordingly, a two-step screening has been recommended^(5)^. In the first step, blood glucose (fasting or random) level and HbA1C measurements are performed during the first prenatal visit, and women who are found to have high risk for diabetes are diagnosed with “overt diabetes” instead of gestational diabetes using standard criteria. The second-step screening strategy of IADPSG is as follows:

- Perform 75 grams OGTT * (as to include fasting, 1-hour, and 2-hour plasma glucose levels between 24-28 weeks of gestation for pregnant women who were not previously diagnosed with overt diabetes)

*OGTT must be performed in the morning after at least 8 hours of fasting.

- The diagnosis of GDM is established is any plasma glucose measurement is above the following limits:

Fasting ≥ 92 mg/dl (5.1 mmol/lt)

1-hour ≥ 180 mg/dl (10.0 mmol/lt)

2-hour ≥ 153 mg/dl (8.5 mmol/lt)

These new criteria may cause a significant increase in the prevalence of GDM due to the fact that one abnormal value (and not two) is sufficient to diagnose GDM. On the other hand, these criteria will allow more pregnant women to receive “medications” and thereby produce good outcomes for the mother and the baby. The results of follow-up studies using these diagnostic criteria are not currently sufficient, and well-designed clinical studies are warranted.

In one of the first large randomized studies that evaluated the effects of treating mild hyperglycemia during pregnancy on the health status of mother and the baby showed that severe neonatal complications have been reduced and postpartum well-being was positively affected^(6)^.

According to another multicenter randomized study, pregnant women with abnormal OGTT but normal fasting blood glucose (FBG<95 mg/dl or 5.3 mmol/lt) were considered to have mild GDM, and the treatment of these cases did not have significant consequences in terms of severe complications of DM such as hyperbilirubinemia, hypoglycemia, hyperinsulinemia, and birth trauma; however, there was a decrease in the incidences of fetal overgrowth, shoulder dystocia, C/S rates, and hypertensive disorder in the mother^(7)^.

The studies have indicated a linear relationship between maternal hyperglycemia and perinatal outcomes but no point value for increased risk. The treatment of even mild maternal hyperglycemia provide good perinatal outcomes(5). Based on the results of these findings, IADPSG considered it necessary to publish new guidelines for gestational diabetes in order to minimize unfavorable perinatal outcomes. Accordingly, it is recommended to obtain 2-hour glucose level with 75 grams OGTT and consider the patient to have GDM if ≥1 abnormal test result is obtained. However, adoption of these new diagnostic criteria will raise the prevalence of GDM up to 18% in the population^(8)^.

There is still no consensus as to how pregnant women diagnosed with these criteria will be monitored but it is obvious that more stringent follow-up is necessary. It must also be emphasized that 80%-90% of pregnant women recently being diagnosed with these criteria have mild GDM which may only require life style change. In other words, it seems possible to raise a healthy generation with simple measures such as diet and exercise. In cases where this remains inadequate, short and long-term insulin therapy must be planned or the patient must be assessed for the use of oral anti-diabetic medications.

## NEW APPROACHES IN THE TREATMENT OF GESTATIONAL DIABETES

It still remains unclear which treatment option is more appropriate when the women with a known GDM and diabetes become pregnant. The most appropriate option would be tailored treatment program in which diet, oral anti-diabetics and/or insulin therapy are selected according to the needs of individual patient.

As in patients with diabetes, body weight, height, BMI, and waist to hip ratio (WHR) must be obtained prior to treatment in patients with GDM. The main elements of the therapy include education, nutritional therapy, exercise, and medical treatment.

The recommended daily calorie intake is 30 kcal/kg for women with a BMI of 22-25, 24 kcal/kg for women with a BMI of 26-29, and 12-15 kcal/kg for women with a BMI of >30. The recommended diet composition contains 33%-40% complex carbohydrates, 35%-40% fat, and 20% protein. This calorie intake may turn 75%-80% of women with GDM into normoglycemic state^(9)^.

The pregnant women in whom blood glucose control cannot be achieved with exercise and diet regulation must be switched to insulin or oral anti-diabetics. There is also no consensus on when to initiate insulin therapy, which has been reported to reduce the risk of macrosomia and other complications during infancy. There are two approaches for the initiation of insulin therapy one of which requires measurement of fasting glucose concentration >90 mg/dl with two weeks intervals and other approach requires postprandial 1-hour glucose measurement >120 mg/dl. The insulin preparations used in GDM include neutral protamine Hagedorn (NPH) and regular insulin. NPH is a moderate-acting insulin and particularly effective in the presence of high fasting glucose levels^(9)^.

The use of oral anti-diabetics (OAD) during pregnancy is a relatively new practice. In a review of the literature regarding this topic, 12 randomized studies were evaluated, and the effects of the use of oral anti-diabetic agents was investigated on pregnant women with a known diabetes and those with impaired glucose tolerance in the current or previous pregnancy^(10)^. According to this review, the disadvantages of OAD agents include lack of clear evidence for the safety of these agents during pregnancy and controversial efficiency of Glyburide (glibenclamide) and Metformin, the most commonly used OAD agents during pregnancy, in preventing postprandial glycemic peaks observed in type 2 diabetes^(11)^. The advantages of OAD agents include easy administration compared to insulin therapy and better patient compliance with relatively less extensive education. OAD agents also offers the advantage of combination with insulin therapy where oral agents remain insufficient, and oral agents also widen the dose range and reduce the amount of insulin.

In a study conducted in South Africa, no significant difference was found in terms of the rates of fetal abnormality between patients who received OAD agents during pregnancy and patients who were placed on or switched to insulin therapy from diet^(12)^. However, this study found higher rate of perinatal mortality in OAD group compared to insulin therapy group.

In a meta-analysis that compared the use of OAD agents and insulin therapy in the management of GDM, a total of 6 randomized controlled studies comprising 1388 cases were evaluated that investigated glycemic control and maternal and perinatal outcomes. This study found no significant difference between OAD group and the insulin group in terms maternal fasting and postprandial glycemic control. There was no significant relationship between the use of OAD agents and neonatal hypoglycemia, increased birth weight, rate of cesarean section, and the incidence of delivering a large baby, and in terms pregnancy outcomes, use of OAD agents or insulin therapy were not found to be significantly different in achieving glycemic control^(13)^.

## MAJOR ORAL ANTI-DIABETIC AGENTS

**Biguanides:** Metformin falls into this group. These agents decrease peripheral insulin resistance, inhibit gluconeogenesis and reduce plasma triglyceride concentrations^(14,15,16)^. Metformin can pass across the placenta. In a study that compared the use of insulin versus metformin during pregnancy, use of metformin did not result in an increase in perinatal complications, and it was even less prone to cause severe neonatal hypoglycemia and it resulted in lesser maternal weight gain and provided better patient compliance. However, metformin was used between 20 and 34 weeks of gestation in this study. There is not sufficient evidence for the safety of metformin in early periods of pregnancy^(17,18)^. In a study that evaluated 126 infants aged 18 months born to 109 mothers, no significant difference was found between mother who received metformin during pregnancy versus those who did not in terms of motor and social development and growth^(19)^. In another study that compared metformin and insulin therapy, no significant difference was found between the two groups in terms of the rate of perinatal complications. Although 46.3% of the women receiving metformin therapy required addition of insulin therapy, the women rather preferred metformin therapy^(20)^. In a recent randomized study, 47 pregnant women with GDM who received metformin or insulin therapy were evaluated, and metformin group had better daily glycemic control, lesser weight gain neonatal hypoglycemia. The logistic regression analysis showed that the need for additional insulin therapy in metformin group was associated with gestational age at diagnosis and mean glucose level before the initiation of therapy^(21)^.

**Sulfonylureas:** Glyburide (glibenclamide) and Glimepiride fall into this group. These drugs increase insulin secretion and peripheral sensitivity to insulin and decrease hepatic clearance of insulin^(22,23,24,16)^. The primary side effect of these drug is hypoglycemia. The first generation sulfonylureas pass across the placenta. It remains unclear whether or not second generation sulfonylureas such as glyburide can pass across the placenta or cause some effects on the fetus^(11,25,26,27)^. The most frightening side effect of sulfonylureas is the stimulation of fetal hyperinsulinemia^(28)^. In a randomized and controlled study, there was no significant between the two patient groups that received glyburide or insulin therapy due to GDM in terms of delivering babies with macrosomia and LGA^(29)^. It was reported that glyburide was equivalent to insulin therapy in terms of achieving glycemic control and comparable to insulin therapy in terms of maternal and fetal complications^(30,31)^.

The data regarding the use of alpha-glycosidase inhibitors, thiazolidines, meglitinides, and peptide analogues are insufficient and mostly experimental.

## FOLLOW-UP OF A DIABETIC PREGNANT WOMEN

The pregnant women who are known to have diabetes or who are diagnosed to have diabetes in the first visit should be placed on a closer follow-up program. General physical examination in addition to neurological examination and fundoscopic examination must be performed at the first visit. Plasma glucose and HbA1 c levels are obtained, and insulin therapy is initiated if required or the dose is adjusted in existing users, and the efficacy of oral anti-diabetics and insulin therapy is evaluated with the measurement of plasma glucose levels at each antenatal visit. In subsequent weeks (generally after 32 weeks), fetal health status is evaluated with antenatal tests.

## CASES WITH HIGHER RISK OF DEVELOPING GESTATIONAL DIABETES

- Previous delivery of a stillbirth baby, anomalous baby, large for gestational age (>4000 grams) or multiple miscarriages,

- GDM in the previous pregnancy,

- BMI > 27 before pregnancy,

- Age > 35 years,

- Diabetes in one of the first degree relatives,

- Recurrent urinary tract infection or fungal infection during pregnancy is a risk factor for developing GDM.

Furthermore, particular attention must be paid to women,

- having large fetus than gestational week,

- having excessive weight gain during pregnancy,

- having polyhydramnios with an unknown cause,

- IUMF,

- With polyuria, polydipsia or glycosuria for possible GDM that remained unnoticed.

The blood glucose level must be regularly tested in a pregnant women with gestational diabetes, compliance to diet or therapy with OAD agent or insulin must be monitored, and the women must be examined with USG for the development of polyhydramnios during routine controls, and the weight of the fetus must be evaluated. The ideal fasting glucose level is 60-90 mg/dl and postprandial 1-hour glucose level is 120 mg/dl during pregnancy. It is useful to test for blood glucose once in every two weeks until 34 weeks of gestations and then 4-7 times daily (before and after meals and before bed). The follow-up blood glucose must be more stringent in patients who receive insulin therapy and in those with uncontrolled glucose levels. NST and BPP must be performed weekly after 36 weeks of gestation. The mother must be education on how to monitor baby movements, and pregnant women with GDM that require insulin therapy must be monitored in the hospital setting after 38 weeks of gestation. The pregnancies complicated with GDM are not desired to extend beyond 40 weeks, and delivery is induced or cesarean section is performed when applicable, if delivery does not start spontaneously. If natural delivery has been planned, close delivery follow-up is necessary.

All pregnant women who were found to have GDM should undergo OGTT at 6 weeks postpartum. Even if this test returns normal, the patient should be explained for her higher risk of developing diabetes in subsequent pregnancies or later in her life compared to general population.

## CONCLUSION

Gestational diabetes is a condition that complicates significant portion of the pregnancies and having significant consequences on the health status of the mother and the baby. However, there is no universally accepted screening test. IADPSG recommended the use of two-step screening program and 75 grams OGTT. Recent studies showed positive effects of decreasing the diagnostic threshold for GDM on perinatal outcomes; however, this will also increase the rate of GDM up to 18% that will bring significant financial burden and over-diagnosis.

Also, it still remains unclear which treatment option would be more appropriate when the women with a known GDM or diabetes become pregnant. The use of oral anti-diabetic agents during pregnancy is a relatively recent matter of debate. The current reports suggest that metformin and glyburide could be used during pregnancy. The most appropriate approach would be to tailor a treatment program in which diet, oral anti-diabetics and/or insulin therapy are selected according to the needs of an individual patient.
